# Role of inflammation and immunity in vascular calcification: a bibliometric and visual analysis, 2000–2022

**DOI:** 10.3389/fcvm.2023.1258230

**Published:** 2023-10-30

**Authors:** Chen Wang, Qingchun Liang, Siyi He, Jie Zhu, Xiafei Lin, Guanwen Lin, Duozhi Wu, Wenqi Zhang, Zhihua Wang

**Affiliations:** ^1^ Department of Anesthesiology, Hainan General Hospital, Hainan Affiliated Hospital of Hainan Medical University, Haikou, China; ^2^ Department of Anesthesiology, The Third Affiliated Hospital, Southern Medical University, Guangzhou, China

**Keywords:** vascular calcification, inflammation, immunity, extracellular vesicles, bibliometric analysis, HistCite, VOSviewer, CiteSpace

## Abstract

**Background:**

In recent years, a great deal of research has been done on vascular calcification (VC), and inflammation and immunity have been displayed to play important roles in the mechanism of VC. However, to date, no comprehensive or systematic bibliometric analyses have been conducted on this topic.

**Methods:**

Articles and reviews on the roles of inflammation and immunity in VC were obtained from the Web of Science Core Collection on August 5, 2022. Four scientometric software packages—HistCite, CiteSpace, VOSviewer, and R-bibliometrix—were used for the bibliometric and knowledge mapping analyses.

**Results:**

The obtained 1,868 papers were published in 627 academic journals by 9,595 authors of 2,217 institutions from 69 countries. The annual number of publications showed a clear growth trend. The USA and China were the most productive countries. Karolinska Institutet, Harvard University, and the University of Washington were the most active institutions. Stenvinkel P published the most articles, whereas Demer LL received the most citations. *Atherosclerosis* published the most papers, while *Circulation* was the most highly cited journal. The largest cluster among the 22 clusters, based on the analysis of co-citations, was osteo-/chondrogenic transdifferentiation. “Vascular calcification,” “inflammation,” “chronic kidney disease,” and “expression” were the main keywords in the field. The keyword “extracellular vesicle” attracted great attention in recent years with the strongest citation burst.

**Conclusions:**

Osteo-/chondrogenic transdifferentiation is the primary research topic in this field. Extracellular vesicles are expected to become a new research focus for exploring the inflammatory and immune mechanisms of VC.

## 1. Introduction

Vascular calcification (VC), characterized by the pathological deposits of minerals in the vascular system (1), is a highly prevalent vascular pathophenotype associated with major adverse cardiovascular events (2–4). The extent and severity of VC are considered major risk factors for predicting cardiovascular morbidity and mortality (5, 6). While VC is regarded as a natural consequence of aging, it progresses at an accelerated rate in several conditions including atherosclerosis, chronic kidney disease, and inflammatory diseases (7). Once thought to be a passive and degenerative process, VC is now considered to be an active and tightly regulated biological process similar to bone formation (8, 9). Recent research has demonstrated that its main molecular mechanisms include chronic inflammation, calcium and phosphorus metabolism disorders, autophagy, apoptosis, endoplasmic reticulum stress, and mitochondrial dysfunction (10). The research on the potential mechanism of VC has aroused great interest among researchers in this field.

Growing evidence suggests that inflammation and immunity are major contributors to VC (11). High levels of cyclic inflammatory markers such as C-reactive protein, interleukin (IL)-6, and tumor necrosis factor-α (TNF-α) are associated with VC prevalence, progression, and severity (12, 13). Proinflammatory immune cells, especially macrophages, can also promote VC by releasing inflammatory cytokines and extracellular vesicles (EVs) (14). Potential therapeutic targets related to inflammation and immunity have also been widely discussed. For example, the inflammatory regulatory signal Rac family small GTPase 2 prevents VC by inhibiting the expression of Rac family small GTPase 1-dependent macrophage IL-1β (15). Therefore, elucidating the roles of inflammation and immunity in VC is of great importance for its prevention and treatment. However, inflammation and immune-promoting VC have not been fully demonstrated in humans. The immune factors that trigger VC and the subsequent inflammatory events have so far revealed only the tip of the iceberg.

Bibliometrics is an interdisciplinary discipline that conducts a quantitative analysis of all knowledge holders through mathematical and statistical methods. Applying bibliometric methods can help researchers quickly evaluate published research results, grasp hotspots and development trends in a field, and lay the foundation for future research (16). Our previous study employed bibliometric analysis for the first time to identify the research status of VC (17). We found that inflammation was a hotspot in VC research. However, the current research trends and hotspots of inflammation and immunity in VC remain unclear. Therefore, we collected articles on this topic, and performed a bibliometric analysis to help researchers gain a complete picture of this field and direct future experimental decisions. Additionally, we offered interpretations and summaries of the most promising research direction and provided references for scholars who delve into this realm.

## 2. Materials and methods

### 2.1. Sources of data and search strategies

**Figure 1:**
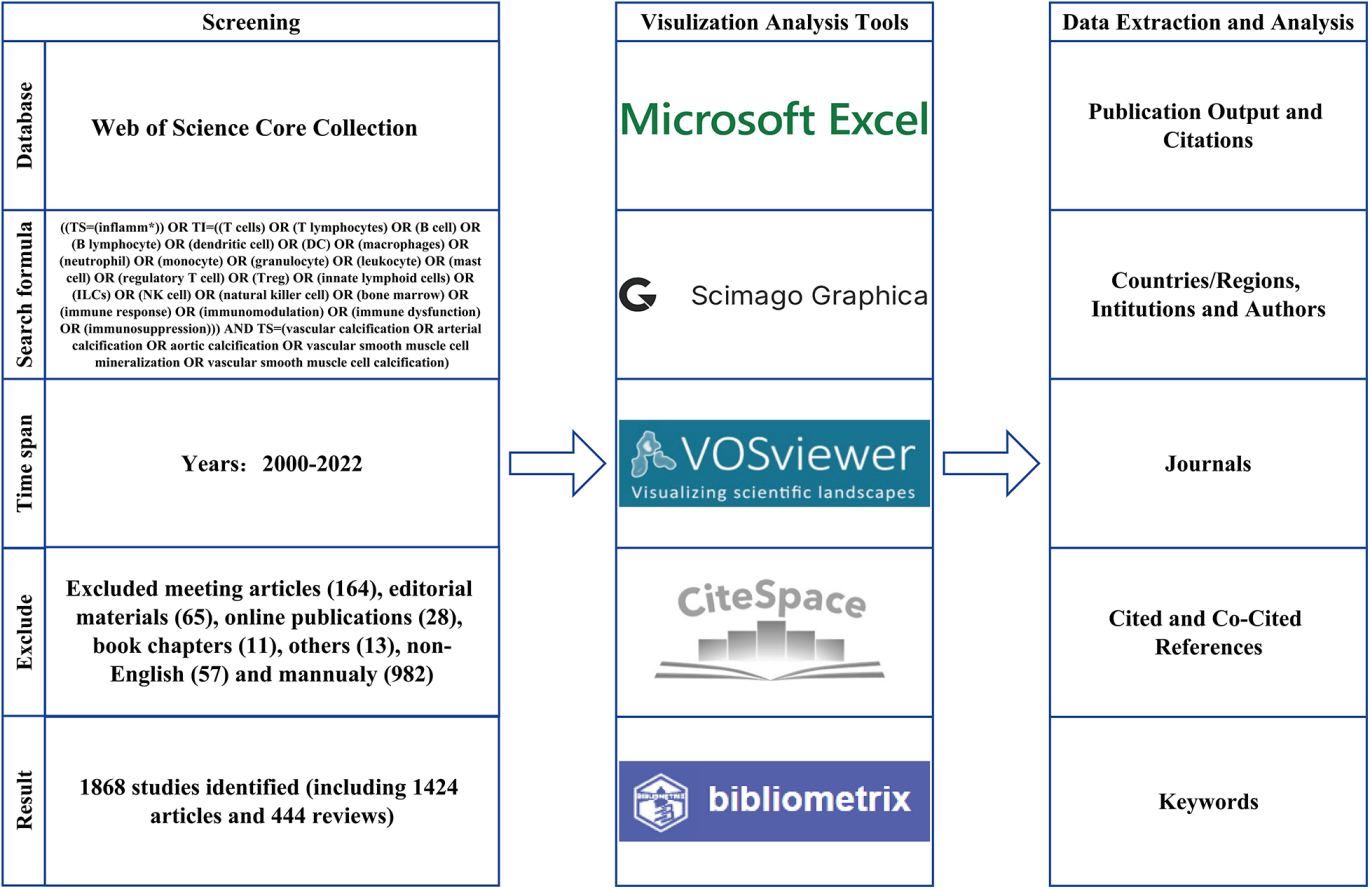
Flowchart of the article screening process and analysis methods.

Bibliometric analysis was conducted using the Web of Science Core Collection (WoSCC). The search formula was set to ([TS = (inflamm*)] OR TI = ((T cells) OR (T lymphocytes) OR (B cell) OR (B lymphocyte) OR (dendritic cell) OR (DC) OR (macrophages) OR (neutrophil) OR (monocyte) OR (granulocyte) OR (leukocyte) OR (mast cell) OR (regulatory T cell) OR (Treg) OR (innate lymphoid cells) OR (ILCs) OR (NK cell) OR (natural killer cell) OR (bone marrow) OR (immune response) OR (immunomodulation) OR (immune dysfunction) OR (immunosuppression))) AND TS = (vascular calcification OR arterial calcification OR aortic calcification OR vascular smooth muscle cell mineralization OR vascular smooth muscle cell calcification) for studies published between January 1, 2000 and August 5, 2022. Complete searches were conducted within one day to avoid errors caused by daily database updates. A total of 1,868 articles were obtained, including only articles and reviews published in English (Figure 1). Two researchers (WC and HSY) selected and recorded the data to ensure that the content was relevant to the topic. All differences were discussed until a consensus was reached.

### 2.2. Statistical analysis

All obtained studies were analyzed and visualized using Microsoft Office Excel 2021, HistCite (version 2.1), Scimago Graphica (version 1.0.24), VOSviewer (version 1.6.18), CiteSpace (version 6.1), and R-bibliometrix (version 4.0).

Microsoft Office Excel 2021 was employed to construct the tables and radar charts.

HistCite (18) was used to determine the number of annual publications, total global citation score (TGCS), total local citation score (TLCS), and top-scoring countries, institutions, authors, and journals. TGCS refers to the total number of citations of publications in the WoSCC database, whereas TLCS refers to the number of citations of publications in the local dataset (finally determined 1,868 publications).

Scimago Graphica (19) was employed to visualize the national cooperation network on a world map or chordal graph. The size of each node indicates the number of publications, while the color of the node or line indicates the strength of the cooperation.

VOSviewer (20) was used to construct the bibliometric network, which included collaborations between countries/regions, institutions, and authors. The node colors indicate different time or clusters, node size represents the number of publications, and line thickness indicates the strength of cooperation.

CiteSpace (21) was used to analyze and visualize knowledge domain and emerging trends, including dual-map overlay and co-cited analysis of journals, co-citation networks, cluster analysis, timeline views of references, co-occurrence analysis of keywords, and citation bursts of references and keywords. The size of the node indicates the total co-citation or occurrence frequency of the elements, and the various colors represent different clusters or years. Lines between nodes indicate co-citations or co-occurrences. Centrality is a metric used to measure the element's significance. When the value of element centrality is greater than 0.1, a purple outer ring is added. Citation bursts represent a large change in citations over time. If an element node has a strong burst, the color of the node is red or pink. Modularity Q and mean silhouette value are two metrics to assess cluster quality. Generally, if modularity Q > 0.3, the cluster structure is significant. And the cluster is considered persuasive if the mean silhouette value > 0.7.

R-bibliometrix (22) was used to analyze the annual increase rate of publications, the publications of top authors over time, and their H-indices and G-indices.

## 3. Results

### 3.1. Analysis of publication outputs and citation

**Figure 2:**
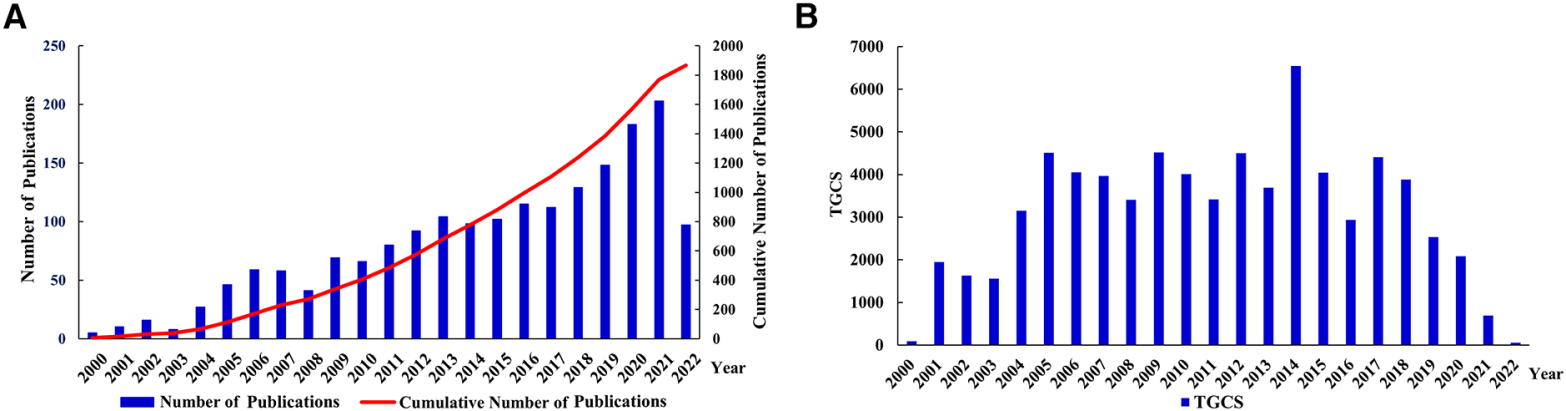
Analysis of publication outputs and citations. (**A**) Annual publications and cumulative publications; (**B**) annual global citations. TGCS, total global citation score.

The search results revealed 1,868 published studies, including 1,424 articles and 444 reviews. Curve fitting analysis (Figure 2A) revealed that, since 2000, the annual publication volume has generally shown an upward trend, with an average annual growth rate of 14.79%. Between 2000 and 2005, fewer than 50 publications on the roles of inflammation and immunity in VC were published annually. Since then, the annual number of publications gradually increased from 59 in 2006 to 191 in 2021. In particular, in the past 5 years, the number of published articles has grown rapidly. As of the search date, these publications have been cited 71,940 times (mean, 38.51). The year 2014 witnessed the highest TGCS of 6,562, indicating a significant level of research excellence during that period. Other relatively highly cited years included 2009 (4,526), 2005 (4,525), and 2012 (4,513). Since 2021 and 2022 were close to the search time (August 5, 2022), their citation frequencies were lower than those in other years (Figure 2B). Annual publications and citations continue to grow, reflecting the rapid development of the field.

### 3.2. Analysis of countries/regions

**Figure 3:**
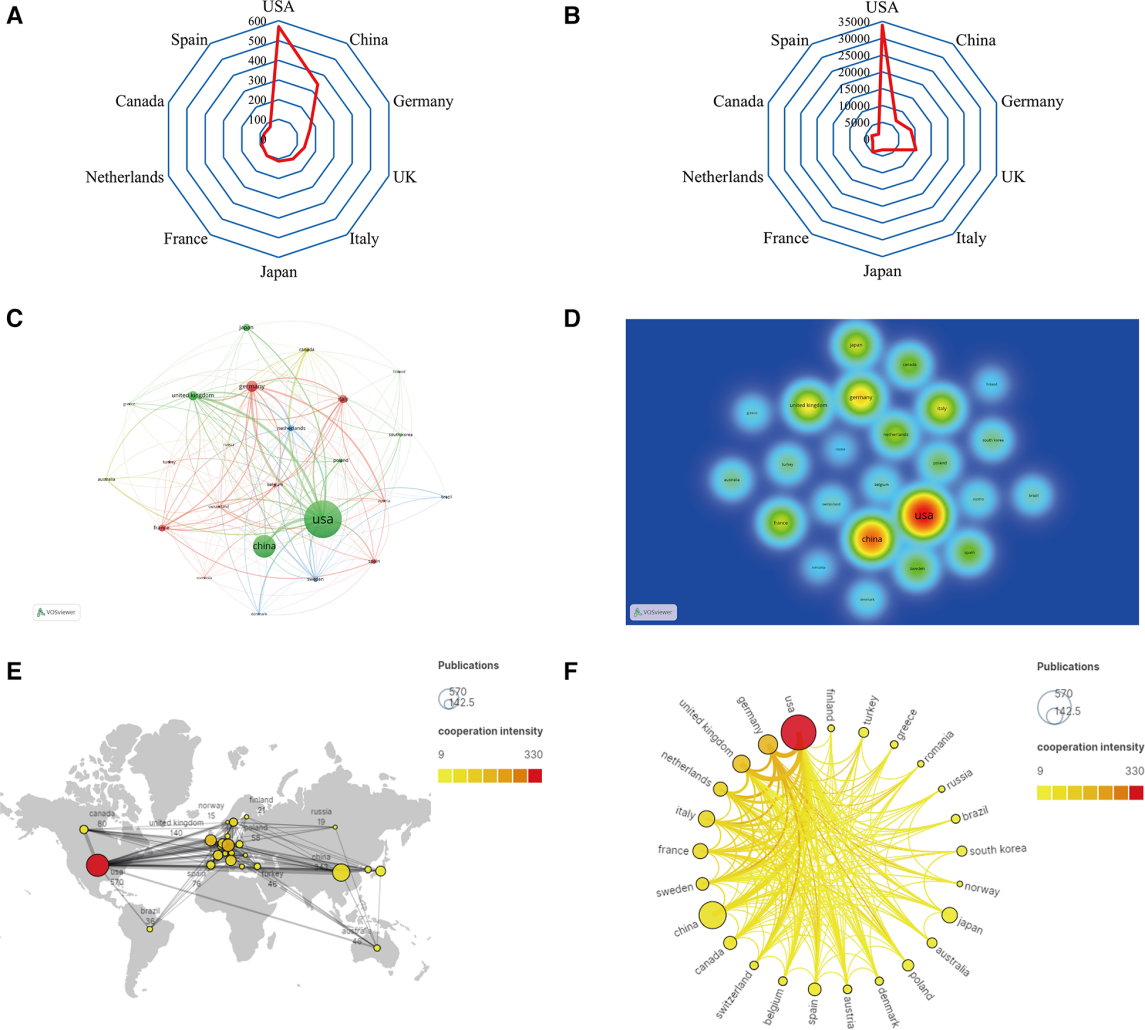
Analysis of countries/regions. (**A**) Radar map of the top 10 productive countries; (**B**) radar map of total global citation score of the top 10 productive countries; (**C**) visual cluster analysis of cooperation among countries; (**D**) density map of cooperation among countries; (**E**) world map of the intensity of cooperation between countries; (**F**) circle diagram of international collaboration between countries.

A total of 69 countries conducted research on the roles of inflammation and immunity in VC. As shown in Figures 3A,C,D and Table 1, the country with the largest number of publications was the USA [570 (30.51%)], followed by China [343 (18.36%)], and Germany [171 (9.15%)]. The USA had the highest TGCS (33,899), followed by the UK (10,322) and Germany (8,665) (Figure 3B). Figure 3E shows that the articles in this field mainly come from North America, Europe, and East Asia. According to the cooperation network between countries (Figures 3C,F), the USA had the highest cooperation intensity (330), indicating that it cooperates more closely with other countries, followed by Germany (179) and the UK (151). Notably, although China had the second-largest number of published articles, its TGCS was only 6,774 and its cooperation intensity was only 76. Thus, Chinese researchers should pay more attention to improving the quality and influence of their papers and strengthening foreign cooperation in the future.

**Table 1:** The top 10 countries with the most publications.

Rank	Country	Publications	TGCS^a^	TLCS^b^	Total link strength
1	USA	570 (30.51%)	33,899	3,677	330
2	China	343 (18.36%)	6,774	682	76
3	Germany	171 (9.15%)	8,665	767	179
4	UK	140 (7.49%)	10,322	961	151
5	Italy	124 (6.64%)	3,918	325	98
6	Japan	111 (5.94%)	3,206	386	28
7	France	104 (5.57%)	4,704	344	88
8	Netherlands	92 (4.93%)	2,921	226	110
9	Canada	80 (4.28%)	3,216	317	65
10	Spain	76 (4.07%)	1,832	158	50

^a^ TGCS, total global citation score.

^b^ TLCS, total local citation score.

### 3.3. Analysis of institutions

A total of 2,217 institutions conducted studies on the roles of inflammation and immunity in VC. Table 2 shows the top 10 institutions with the most published articles. Among them, the Swedish Karolinska Institutet (49) had the highest output, followed by Harvard University (42) and the University of Washington (40). Half of the 10 most productive institutions were located in the USA.

**Table 2:** The top 10 institutions that published the highest number of publications.

Rank	Institution	Country	Publications	TGCS^a^	TLCS^b^
1	Karolinska Institutet	Sweden	49 (2.62%)	1,951	182
2	Harvard University	USA	42 (2.25%)	3,820	564
3	University of Washington	USA	40 (2.14%)	3,629	299
4	University of California Los Angeles	USA	39 (2.09%)	4,614	841
5	Harvard Medical School	USA	35 (1.87%)	818	102
6	Maastricht University	Netherlands	33 (1.77%)	590	57
7	University of Edinburgh	UK	31 (1.66%)	3,188	368
8	University of Cambridge	UK	27 (1.45%)	3,090	326
9	Huazhong University Science and Technology	China	27 (1.45%)	480	80
10	Brigham and Women's Hospital	USA	24 (1.28%)	1,792	205

^a^ TGCS, total global citation score.

^b^ TLCS, total local citation score.

**Figure 4:**
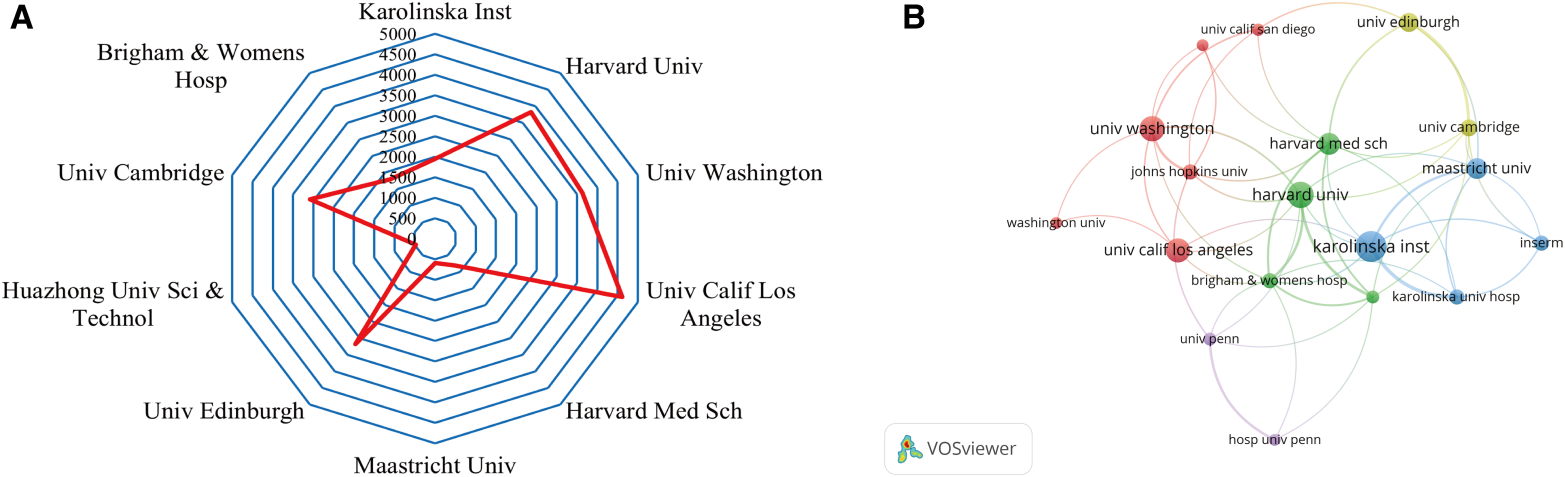
Analysis of institutions. (**A**) Radar map of the top 10 productive institutions; (**B**) visual cluster analysis of cooperation among institutions.

The top three institutions with the highest TGCS were the University of California Los Angeles (4,614), Harvard University (3,820) and the University of Washington (3,629) (Table 2, Figure 4A). According to Figure 4B, institutions with more than or equal to 17 publications were used to construct the cooperation map. According to the cooperation map, cooperation between institutions was relatively close. For example, Harvard University has close cooperation with the University of California Los Angeles, Karolinska Institutet, Brigham and Women's Hospital and Maastricht University, etc. In the future, cooperation between institutions should continue to be strengthened, and concerted efforts should be made to advance the development of this field.

### 3.4. Analysis of authors

**Figure 5:**
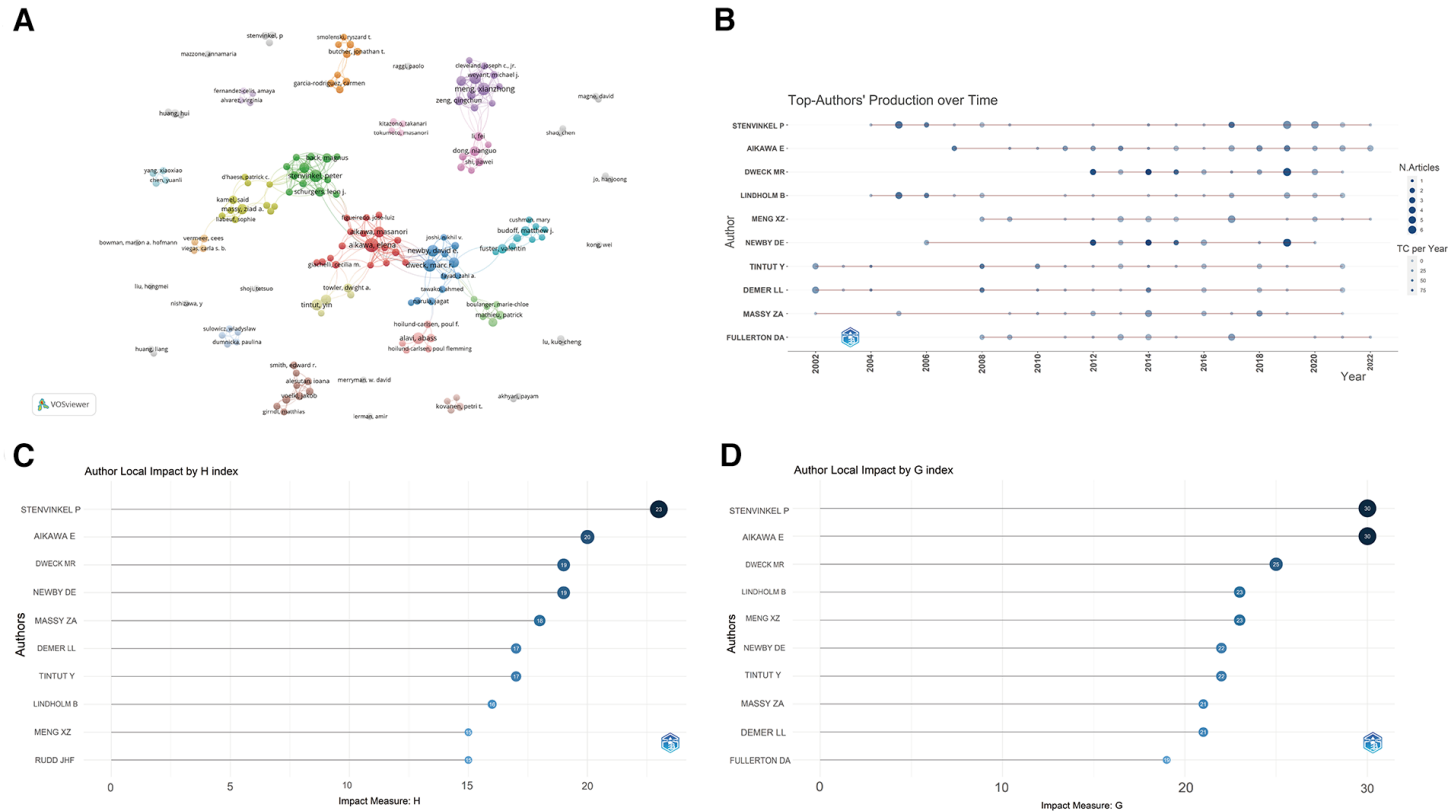
Analysis of authors. (**A**) Cooperation network of authors; (**B**) the top authors’ production over time; (**C**) author local impact by H-index; (**D**) author local impact by G-index.

A total of 9,595 authors engaged in research and published articles in this field. The top 10 most prolific authors were listed in Table 3. Among these productive authors, Stenvinkel P published the most articles (32), followed by Aikawa E (31) and Dweck MR (25), respectively. Demer LL was the most cited author (3,119), followed by Dweck MR (3,018) and Newby DE (2,957). As shown in Figure 5A, Aikawa E closely cooperated with Aikawa M, Rogers MA, and Body SC. In addition, Stenvinkel P frequently collaborated with Lindholm B, Back M, and Schurgers LJ. Close cooperation was also observed between clusters such as Xianzhong Meng and Fei Li, Dweck MR and Aikawa E.

**Table 3:** The top 10 productive authors.

Rank	Author	Publications	Country	Institution	TGCS^a^	TLCS^b^
1	Stenvinkel P	32 (1.71%)	Sweden	Karolinska Institutet	2,658	190
2	Aikawa E	31 (1.65%)	USA	Harvard University	2,825	567
3	Dweck MR	25 (1.33%)	UK	University of Cambridge	3,018	359
4	Meng XZ	24 (1.28%)	USA	Children's Hospital Colorado	822	260
5	Lindholm B	23 (1.23%)	Sweden	Karolinska Institutet	1,949	142
6	Newby DE	22 (1.17%)	UK	University of Edinburgh	2,957	337
7	Tintut Y	22 (1.17%)	USA	University of California	2,908	568
8	Demer LL	21 (1.12%)	USA	University of California	3,119	603
9	Massy ZA	21 (1.12%)	France	Assistance Publique Hopitaux Paris	1,106	77
10	Fullerton DA	20 (1.07%)	USA	University of Colorado	775	256

^a^ TGCS, total global citation score.

^b^ TLCS, total local citation score.

The timeline of the authors' publications was also plotted (Figure 5B). Among the top 10 prolific authors, Demer LL, Tintut Y, and Massy ZA have been working in this field for at least 20 years (since 2002), while other authors have been engaged in publication for the roles of inflammation and immunity in VC since 2004. Although Dweck MR has only been working in the field for 10 years, he has published many high-impact articles. Furthermore, the G and H indices of all authors were calculated, Stenvinkel P and Aikawa E had the highest number and level of academic output (Figures 5C,D). Based on the above analysis, they have great academic influence in this field.

### 3.5. Analysis of journals

**Figure 6:**
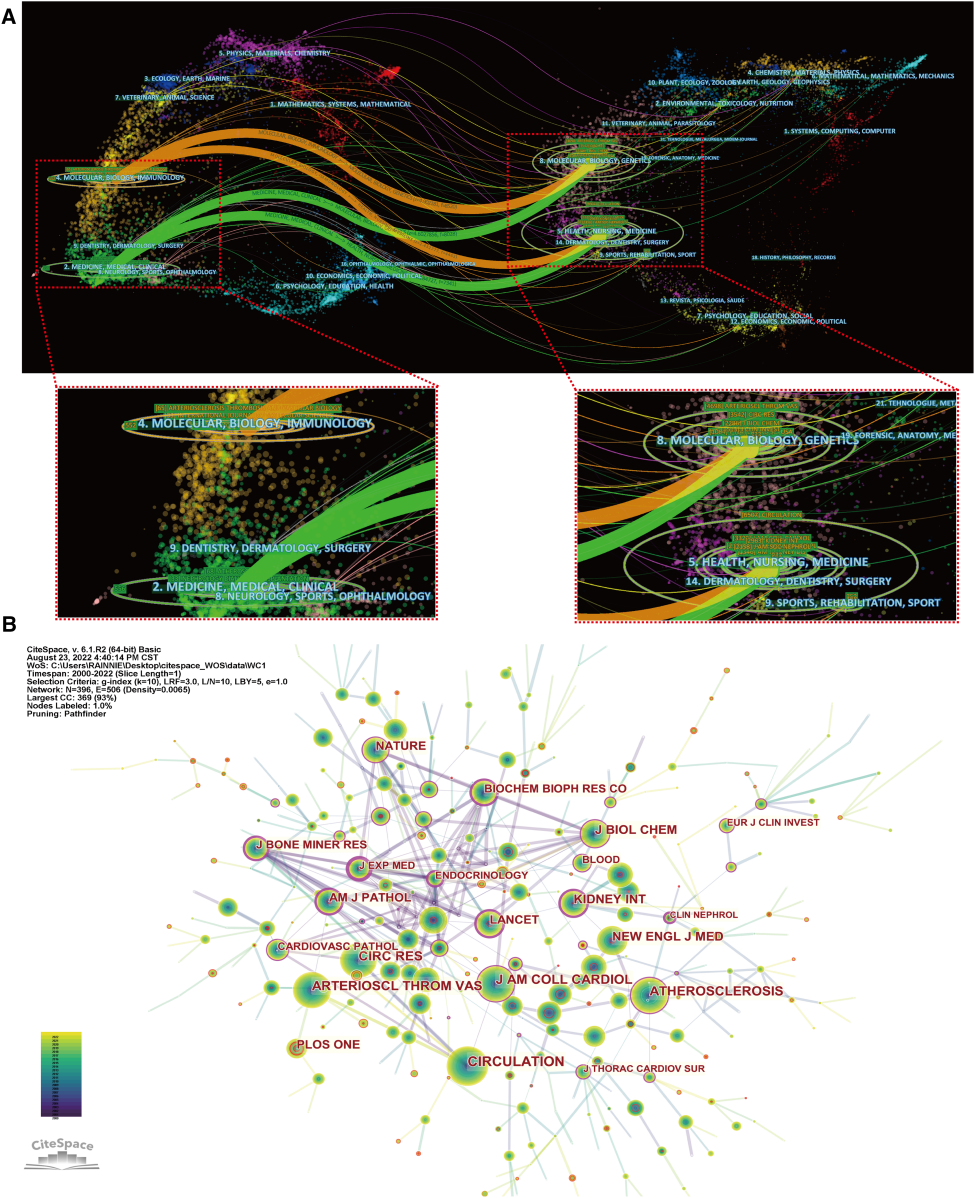
Analysis of journals. (**A**) The dual-map overlay of journals; (**B**) visualization of co-cited journals.

Figure 6A shows a dual-map overlay of journals by topic distribution. The citing journal distribution is shown on the left and the cited journal distribution is shown on the right. The labels show the topics covered by the journal, while the colored paths represent the citation relationships (23). Four main pathways were identified in this study. Most papers were published in journals belonging to the fields of molecular biology, immunology, medicine, and clinical practice, and they mainly cited journals in the fields of molecular biology, genetics, health, nursing, and medicine.

A total of 627 journals accepted studies on the roles of inflammation and immunity in VC. Table 4 shows the top 10 core journals. The journal with the most published papers in this field was *Atherosclerosis* (68), followed by *Atherosclerosis Thrombosis and Vascular Biology* (65) and *Nephrology Dialysis Transplantation* (33), respectively. Among the top 10 journals with the highest number of publications, *Circulation* had the highest impact factor (IF) of 39.918. An analysis of co-cited academic journals revealed that *Circulation* (1,523) had the most co-citations, followed by *Atherosclerosis Thrombosis and Vascular Biology* (1,281) and *Circulation Research* (1,069) (Figure 6B). Among the top 10 most frequently cited academic journals, *New England Journal of Medicine* had the highest IF of 176.079. Ninety percent of the top 10 most co-cited academic journals were classified as Q1 (Table 5). In addition, the journal with the highest centrality was *Journal of Experimental Medicine*. The above analysis shows that they have a strong academic reputation and influence in this field.

**Table 4:** The top 10 journals by the number of publications.

Rank	Journal	Publications	TGCS^a^	TLCS^b^	JCR partitions	Impact factor (2021)
1	Atherosclerosis	68 (3.64%)	2,750	247	Q1	6.847
2	Atherosclerosis Thrombosis and Vascular Biology	65 (3.48%)	5,446	707	Q1	10.514
3	Nephrology Dialysis Transplantation	33 (1.77%)	2,077	226	Q1	7.186
4	International Journal of Molecular Sciences	33 (1.77%)	388	0	Q1	6.208
5	Plos One	30 (1.61%)	861	0	Q2	3.752
6	Circulation Research	28 (1.50%)	5,286	569	Q1	23.213
7	Frontiers in Cardiovascular Medcine	28 (1.50%)	135	0	Q2	5.846
8	Circulation	24 (1.28%)	5,472	877	Q1	39.918
9	Kidney International	19 (1.02%)	2,185	172	Q1	18.998
10	Scientific Reports	19 (1.02%)	280	0	Q2	4.996

^a^ TGCS, total global citation score.

^b^ TLCS, total local citation score.

**Table 5:** The top 10 journals by co-citation and centrality.

Rank	Journal	Co-citations	JCR partitions	Impact factor (2021)	Journal	Centrality	JCR partitions	Impact factor (2021)
1	Circulation	1,523	Q1	39.918	Journal of Experimental Medicine	0.43	Q1	17.579
2	Atherosclerosis Thrombosis and Vascular Biology	1,281	Q1	10.514	Kidney International	0.35	Q1	18.998
3	Circulation Research	1,069	Q1	23.213	Biochemical and Biophysical Research Communications	0.35	Q3	3.322
4	Journal of the American College of Cardiology	1,044	Q1	27.203	Lancet	0.32	Q1	202.731
5	Atherosclerosis	983	Q1	6.39	Journal of Bone and Mineral Research	0.32	Q1	6.39
6	New England Journal of Medicine	920	Q1	176.079	Clinical Nephrology	0.25	Q4	1.243
7	Journal of Biological Chemistry	759	Q2	5.486	American Journal of Pathology	0.24	Q1	5.77
8	Journal of Clinical Investigation	738	Q1	19.456	Endocrinology	0.2	Q2	5.051
9	Kidney International	699	Q1	18.998	Journal of Biological Chemistry	0.19	Q2	5.486
10	Journal of the American Society of Nephrology	653	Q1	14.978	Genes & Development	0.19	Q1	12.89

### 3.6. Analysis of cited and co-cited references

Analyzing the cited literature provides insight into the fundamentals and context of the field. The most cited study was authored by Mohler et al. in 2001, who detected the pattern of inflammatory cell infiltration and the types of infiltrating cells in calcified heart valves and found that heterotopic ossification was common in end-stage valvular heart disease (24). According to Table 6, the most co-cited study was authored by Durham et al. in 2018, who reviewed the role of vascular smooth muscle cells (VSMCs) in driving VC in terms of inflammation and oxidative stress. In addition, the authors pointed out that the transdifferentiation of phenotype and the release of EVs from macrophage lineage are the focus of the future exploration of the treatment of VC (9). It is worth mentioning that the article by Al-Aly Z et al., although not the most co-cited study, has encouraged subsequent researchers to explore the inflammatory and immune mechanisms of VC. They used an Ldlr**^-/-^** mouse model to demonstrate that TNF-α enhances aortic Msx2-Wnt pathways that contribute to aortic calcification in type II diabetes (25).

**Table 6:** The top 10 co-cited references.

Rank	First author	Year	Journal	Co-citations	Title
1	Durham AL	2018	Cardiovascular Research	108	Role of smooth muscle cells in vascular calcification: implications in atherosclerosis and arterial stiffness
2	Ketteler M	2003	Lancet	63	Association of low fetuin-A (AHSG) concentrations in serum with cardiovascular mortality in patients on dialysis: a cross-sectional study
3	Ridker PM	2017	New England Journal of Medicine	62	Anti-inflammatory therapy with canakinumab for atherosclerotic disease
4	Joshi NV	2014	Lancet	61	^1^⁸F-fluoride positron emission tomography for identification of ruptured and high-risk coronary atherosclerotic plaques: a prospective clinical trial
5	Schafer C	2003	Journal of Clinical Investigation	60	The serum protein α2–Heremans-Schmid glycoprotein/fetuin-A is a systemically acting inhibitor of ectopic calcification
6	Irkle A	2015	Nature Communications	58	Identifying active vascular microcalcification by 18F-sodium fluoride positron emission tomography
7	Mohler ER	2001	Circulation	57	Bone formation and inflammation in cardiac valves
8	Lindman BR	2016	Nature Reviews Disease Primers	55	Calcific aortic stenosis
9	Al-Aly Z	2007	Arteriosclerosis Thrombosis and Vascular Biology	50	Aortic Msx2-Wnt calcification cascade is regulated by TNF-α–dependent signals in diabetic Ldlr**^-/-^** mice
10	London GM	2003	Nephrology Dialysis Transplantation	48	Arterial media calcification in end-stage renal disease: impact on all-cause and cardiovascular mortality

**Figure 7:**
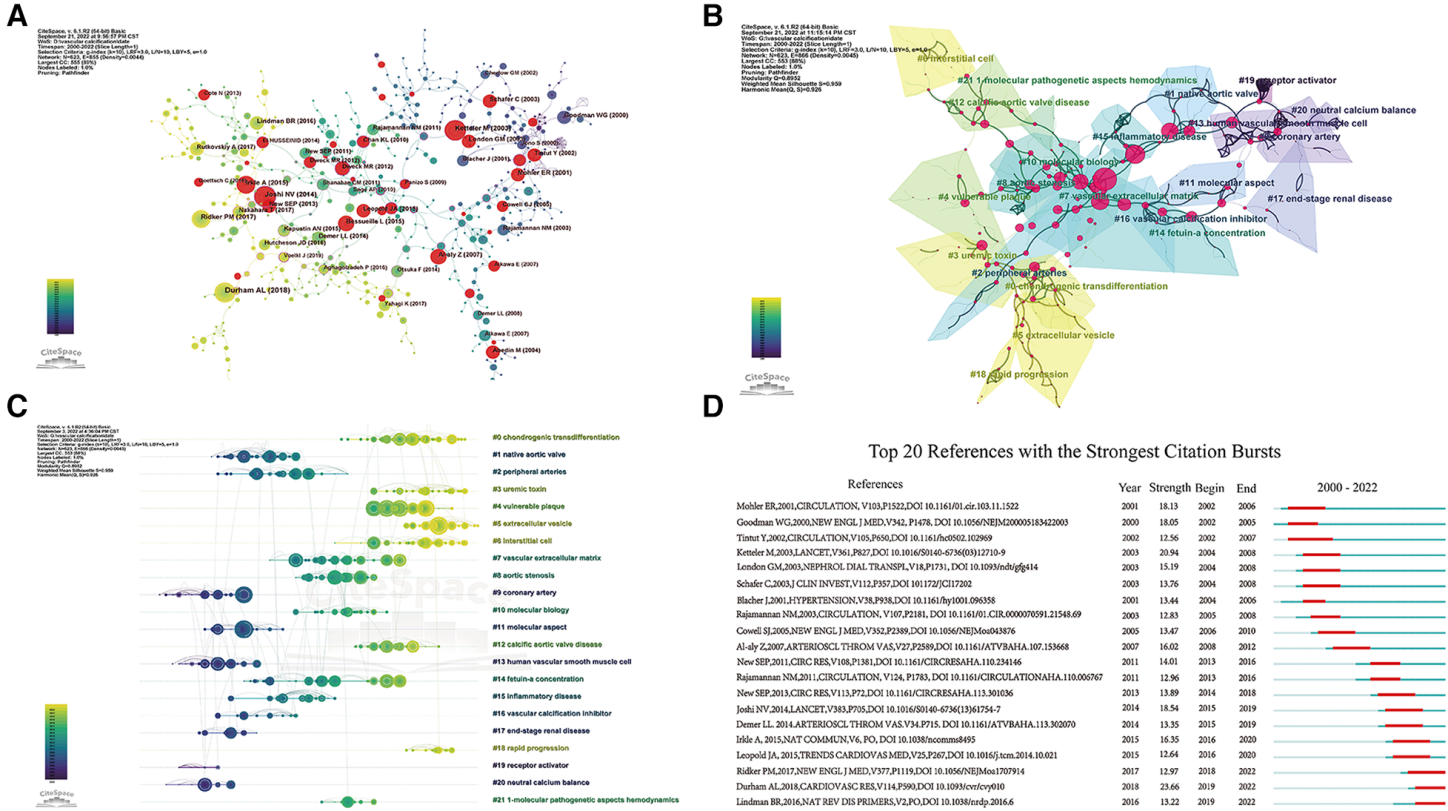
Analysis of co-cited references. (**A**) The co-cited references citation network; (**B**) cluster analysis of co-cited references; (**C**) timeline distribution of the 22 clusters; (**D**) top 20 references with the strongest citation bursts.

In addition, we must consider the effect of time on citations: papers published earlier are often cited more often than papers published later. Therefore, we constructed a co-cited network of references (Figure 7A) that were subsequently clustered and marked the core pathways of transitions among clusters (Figure 7B). The modularity Q was 0.8952, each silhouette value > 0.7, suggesting that the results of the cluster analysis were credible. Cluster labels were extracted and named using the log-likelihood ratio algorithm. A total of 22 clusters were plotted, mainly including “chondrogenic transdifferentiation,” “extracellular vesicle,” and “fetuin-a concentration.” Among the 22 clusters, cluster #0 (“osteo-/chondrogenic transdifferentiation”) was the largest. We then performed a timeline analysis of the clusters (Figure 7C). Obviously, EVs have recently attracted researchers' attention. Finally, through a citation burst analysis, 20 studies with the strongest citation bursts were screened out (Figure 7D). The timeline consists of red and green lines, with red indicating periods of high citation bursts and green indicating low citation bursts. It is worth noting that the article written by Durham et al. (9) had the highest citation burst strength (23.66). Durham (9) and Lindman (2016) had the highest citation burst strengths from 2019 to 2022.

### 3.7. Analysis of keywords

**Figure 8:**
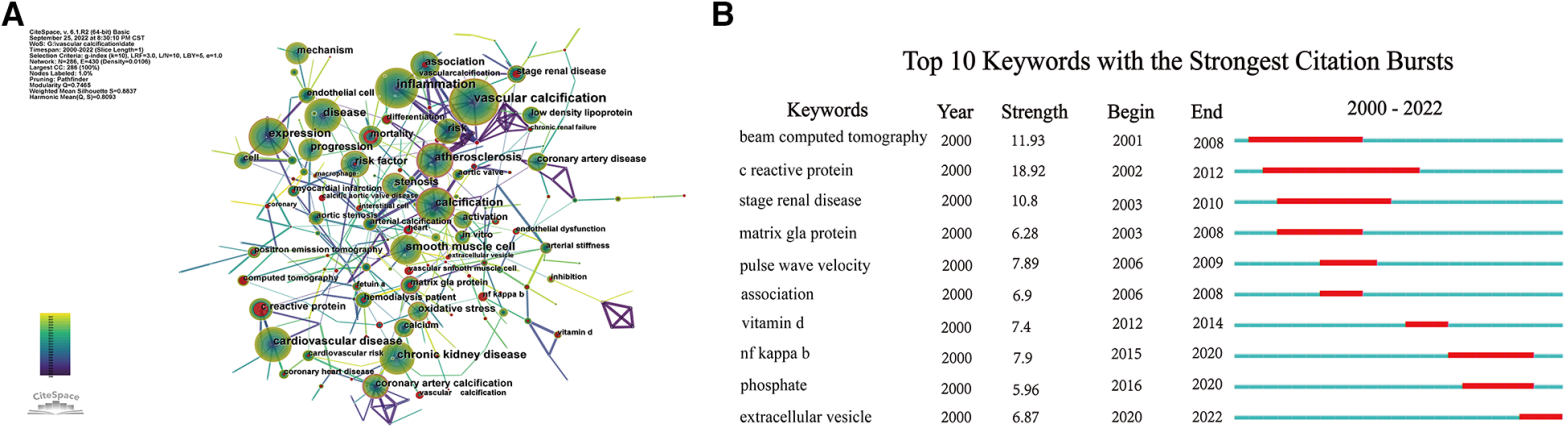
Analysis of keywords. (**A**) Network map of keywords; (**B**) top 10 keywords with the strongest citation bursts.

The indexing of keywords is convenient for understanding the main content of a paper, so analyzing keywords can rapidly identify the hotspots and frontiers in a certain field. Through the co-occurrence network of keywords, “vascular calcification,” “inflammation,” “chronic kidney disease,” and “expression” were the most frequently occurring keywords (Figure 8A). Keyword citation burst detection is a concise and effective means of displaying cutting-edge research trends and future academic hotspots in a certain field. Among the top 10 keywords with the highest citation explosion, “C-reactive protein” attracted the most attention from fellow researchers over the past 22 years (Figure 8B). In addition, “NF kappa B” and “phosphate” received greater attention from 2015 to 2020, while “extracellular vesicle” became a new research hotspot in 2020.

## 4. Discussion

In the current study, we visualized the knowledge domain and general trends of research on the roles of inflammation and immunity in VC. Annual publications in this field generally showed an increasing trend over the past two decades, particularly since 2018. The annual mean growth rate of published papers was 14.79%. Therefore, it is anticipated that the research in this field will continue to expand, leading to substantial growth in both the number of publications and citations in the next few years.

The quantity and quality of papers published in a certain research field are considered to be important indicators for evaluating the scientific research level of countries, institutions, and authors. Analysis of countries, institutions, and authors can help us identify where the excellent scientific research force in this field is located. The USA produced the most publications and had the highest TGCS and cooperation intensity, which could be explained by several factors. For example, the USA had the most institutions and authors with the highest TGCS as well as the most cited references. Interestingly, although China also had the second-largest number of publications, its articles received fewer citations. This situation may be related to the fact that Chinese scientific research cooperation mainly occurred within its territory and scientific research conditions were poor in the past few years. In recent years, China has introduced relevant policies to support scientific research (26); thus, it is believed that the level and quality of scientific research in China will improve. Moreover, Stenvinkel P from Sweden was the most prolific author, and his institution, Karolinska Institutet, was the most productive. At the same time, his G and H indices were the highest among all authors, so his articles had a high value. His team mainly focused on the mechanisms of VC associated with inflammation and immunity, and anti-inflammatory treatment strategies, such as the G-protein coupled receptor ChemR23 that can induce inflammation to subside, Fetuin-A as a circulating calcification inhibitor, and alkaline phosphatase as a target of VC therapy (27–29).

Journals serve as a pivotal medium for the dissemination of scholarly literature; therefore, publishing in reputable journals can significantly enhance the visibility and impact of authors and their works (30). *Atherosclerosis* is a famous journal that publishes the most articles in this field, and authors may consider selecting it for submission. IF refers to the frequency of citations of articles in a journal in a specific year or period and is an important indicator for measuring academic journals. Among the top 10 journals for co-citation, 80% had an IF > 10. Therefore, in this field, article quality and research level are excellent. Additionally, academic journals with high co-citation rankings can serve as high-quality sources of reference for our manuscript. According to the analysis, the journal with the highest co-citations was *Circulation*. *Circulation* is among the top journals in cardiovascular academia, so it undoubtedly attaches importance to the research on the role of inflammation and immunity in VC. Moreover, based on the dual-map overlay of journals, current research is mainly concentrated in the fields of basic medicine and clinical medicine, while it is necessary to further expand the field to promote its development.

According to co-citation cluster analysis, osteo-/chondrogenic transdifferentiation is the topic of the greatest concern to researchers in this field. Osteo-/chondrogenic of VSMCs is promoted by pro-inflammatory pathways activated under high Pi conditions, enhancing VC through various mechanisms (31). The key step in enhancing osteo-/chondrogenic transdifferentiation of VSMCs is that Pi exposure activates the proinflammatory transcription factor nuclear factor κ-light-chain-enhancer of activated B cells (32). Recently, extensive studies have been devoted to regulating osteo-/chondrogenic transdifferentiation and calcification of VSMCs by regulating nuclear factor κ-light-chain-enhancer of activated B cells signaling (33, 34). As shown in Figure 7B, Cluster 0# (“osteo-/chondrogenic transdifferentiation”) and 5# (“extracellular vesicles”) partially overlap. The reason is that increased uptake of pro-calcific EVs may contribute to osteo-/chondrogenic transdifferentiation of VSMCs, while these cells also release EVs that promote VC (31).

**Figure 9:**
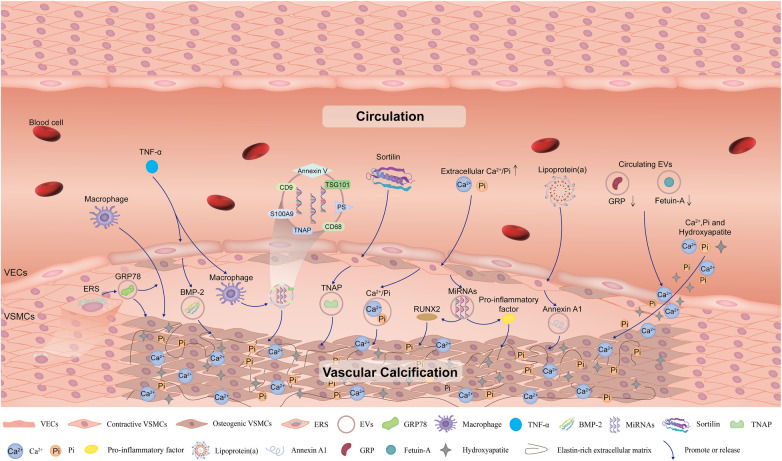
Schematic diagram of the impact of extracellular vesicles on vascular calcification. Extracellular vesicles derived from VSMCs, VECs, and macrophages under specific conditions such as high levels of Ca^2+^/Pi, inflammation and endoplasmic reticulum stress, promote VC. In addition, circulating extracellular vesicles also contribute to mineral deposits in the vessels and promote VC. VC, vascular calcification; VSMCs, vascular smooth muscle cells; VECs, vascular endothelial cells; ERS, endoplasmic reticulum stress; GRP78, glucose-regulated protein 78; TNF-α, tumor necrosis factor-α; BMP-2, bone morphogenetic protein-2; TNAP, tissue non-specific alkaline phosphatase; PS, phosphatidylserine; EVs, extracellular vesicles; GRP, Gla-rich protein.

From the timeline analysis of the clusters and citation bursts of references and keywords, extracellular vesicles have recently attracted much attention. EVs, membrane-enclosed vesicles, which can transfer nucleic acids, protein cargo and metabolites to specific recipient cells in order to modify the phenotype of these cells (35). EVs participate in physiological and pathological processes such as inflammation, immunity, and vasoactive responses, and advance VC (36). EVs are mainly derived from VSMCs, vascular endothelial cells (VECs), macrophages, and circulation, which promote VC through paracrine and endocrine pathways. EVs derived from VSMCs, VECs and macrophages act primarily locally, while circulating EVs from unknown donor cells act remotely (Figure 9). In the next two paragraphs, we mainly describe how EVs regulate VC.

It has been widely reported that VSMCs, VECs, and macrophages release EVs into the calcified microenvironment in a paracrine manner and finally play important roles in VC. In VSMCs, EVs induced by increased expression of glucose-regulated protein 78 and transcription factor 4 under endoplasmic reticulum stress conditions attract inflammatory cells and promote VC (37, 38). Moreover, in the inflammatory environment of osteogenic differentiation of VSMCs, sortilin adjusted the loading of the calcification protein tissue nonspecific alkaline phosphatase (TNAP) into VSMC-derived EVs, promoting inflammation-driven VC (39). Furthermore, the release of VECs-derived EVs with high bone morphogenetic protein-2 levels are stimulated by inflammatory factors such as TNF-α, which can contribute to osteogenic differentiation and VC (40). In addition, under pathological conditions of chronic kidney disease, the increase in extracellular Ca2+/Pi concentration leads to the release of EVs with high levels of these ions by VECs to induce VC (41). These EVs tend to aggregate and form microcalcifications in collagen-sparse regions when released into the extracellular matrix. Subsequently, microcalcifications accumulate into large calcifications and gradually form mature minerals (42).

EVs with calcification potential may also contain unbalanced and dysfunctional miRNAs, which induce gene expression and protein synthesis of osteogenic markers, such as RUNX family transcription factor 2 and proinflammatory factors, and ultimately lead to VC (41). Hence, miRNAs in EVs may serve as intervention targets for VC. Moreover, the release of annexin-rich EVs is stimulated by the uptake of pro-inflammatory lipoprotein(a) by VSMCs, especially microvesicles (CD29^+^/tetraspanin− EVs) trapped in the collagenous extracellular matrix and form microcalcifications that eventually contribute to VC (43). In addition, macrophage-derived EVs promote osteogenic differentiation of VSMCs and lead to VC, especially in an inflammatory environment (i.e., TNF-α) (44, 45). EVs secreted by macrophages contain the tetraspanin exosomal markers CD9, CD68, and TSG101 (46). At the same time, TNAP is loaded into macrophage-derived EVs and plays a key role in inflammation-driven osteogenic calcification in atherosclerotic plaques (47). S100A9 is a proinflammatory and prothrombotic factor that plays an important role in VC (48). S100A9 mediates the mineralization of macrophage-derived calcified EVs by interacting with annexin V, forming a phosphatidylserine-annexin V-S100A9 membrane complex as a nucleation site for hydroxyapatite (44). Finally, the accumulation of these EVs increases the mineralization of vascular calcified plaques (41).

Circulating EVs can be taken up by VSMCs, promoting VC through calcification/osteogenic differentiation and inflammation. Both Gla-rich protein and fetuin-A are inhibitors of VC; Gla-rich protein also has anti-inflammatory effects on immune cells by reducing proinflammatory responses (49, 50). Therefore, the circulating EVs containing these proteins play crucial roles in the development of VC. For example, circulating EVs with lower levels of Gla-rich protein and fetuin-A promote the deposition of calcium, phosphorus ions and hydroxyapatite crystals by increasing osteogenic differentiation and inflammation in the VSMCs (51).

Most current studies of immune cells in EV-mediated VC have focused on macrophages, but some recent studies have shown that other innate immune cells such as neutrophils and dendritic cells can also release EVs to promote vascular remodeling. Neutrophil microvesicles enhance NF-κB by delivering miR-155, promoting vascular inflammation and atherosclerosis (52). In addition, dendritic cell exosomes contribute to endothelial inflammation and atherosclerosis via the membrane TNF-a- mediated NF-κB pathway (53). However, whether EVs derived from neutrophils and dendritic cells lead to VC remains unclear, and the molecular mechanism requires further exploration. In addition, the source of circulating EVs requires addressing in the future.

## 5. Limitations

In the present study, we used powerful bibliometric software to systematically display the current status of research on inflammation and immunity in VC. However, our study still had some limitations. First, as a result of the limitations of the current bibliometric software, we only analyzed the data downloaded from the WoSCC database, therefore, we could miss some data only included in other databases such as Pubmed and Scopus databases. Second, only articles and reviews in English were included in this study, while online publications, edited materials, meeting articles, book chapters, and non-English articles were excluded. Finally, due to the update of databases and software, the current knowledge map of this field is temporary, and needs to be updated in the future.

## 6. Conclusions

Here we analyzed the knowledge base, hotspots, and future trends of research on the roles of inflammation and immunity in VC over the past two decades using HistCite, VOSviewer, CiteSpace, and R-bibliometrix. The USA contributes the most to this field. Karolinska Institutet, *Atherosclerosis* and Stenvinkel P are the institution, journal and author with the most publications, respectively. However, the institution, journal and author that have the most citations are the University of California Los Angeles, *Circulation,* and Demer LL. The article “Role of smooth muscle cells in VC: Implications in atherosclerosis and arterial stiffness,” written by Durham AL, is the most commonly cited. In this field, osteo-/chondrogenic transdifferentiation is a main research topic that has received much attention. Additionally, according to our knowledge map analysis, we believe that EVs will become a focus of future research in this field.

## Data Availability

The original contributions presented in the study are included in the article/Supplementary Material, further inquiries can be directed to the corresponding authors.
